# Clinical and Physiological Characterization of Elevated Plasma Glucagon-Like Peptide-1 Levels (Hyperglipemia) in a Dipeptidyl Peptidase IV Mutation Carrier

**DOI:** 10.3389/fendo.2018.00062

**Published:** 2018-03-05

**Authors:** Dandan Zhao, Shaoqian Zhao, Xiao Wang, Mingbo Su, Wen Liu, Qinyun Ma, Jie Hong, Weiqiong Gu, Jingya Li, Ruixin Liu, Guang Ning, Jiqiu Wang, Yifei Zhang

**Affiliations:** ^1^Department of Endocrinology and Metabolism, China National Research Center for Metabolic Diseases, National Key Laboratory for Medical Genomes, Ruijin Hospital, Shanghai Jiao Tong University School of Medicine (SJTUSM), Shanghai, China; ^2^National Center for Drug Screening, Shanghai Institute of Material Medical (SIMM), Chinese Academy of Science (CAS), Shanghai, China; ^3^Institute of Health Sciences, Shanghai Institutes for Biological Sciences (SIBS), Chinese Academy of Sciences (CAS), Shanghai Jiao Tong University School of Medicine (SJTUSM), Shanghai, China

**Keywords:** dipeptidyl peptidase IV, glucagon-like peptide-1, β-cell function, type 2 diabetes, incretin effect

## Abstract

The clinical application of dipeptidyl peptidase IV inhibitors (DPP4i) increasing active glucagon-like peptide-1 (AGLP-1) levels has been linked to pancreatitis, pancreatic tumors, and cardiovascular events. However, *DPP4* mutations in humans or the long-term outcomes of high glucagon-like peptide-1 (GLP-1) level exposure have not been reported. A trio family with a proband showing an extremely high AGLP-1 level [defined here as hyperglipemia (hyper-glucagon-like peptide-1-emia)] were conducted whole-exome sequencing for potential pathogenic genetic defects. One novel *DPP4* mutation, p.V486M (c.1456 G>A), was identified in the proband and showed damaged enzymatic activity of DPP4. *Ex vivo* functional study further showed that the serum from the proband markedly enhanced insulin production of primary rat islet cells. Furthermore, V486M variant and another eight *DPP4* variants were identified in our in-home database and seven showed decreased enzymatic activities than wild-type DPP4, consistent with their alterations in their protein expression levels. Of note, the levels of glucose, lipids, and tumor markers (especially for CA15-3 and CA125), increased gradually in the proband during a 4-year follow-up period, although no abnormal physical symptoms or imaging results were observed at present. The other two old carriers in the pedigree both had type 2 diabetes, and one of them also had hyperlipidemia and myocarditis. We first identified hyperglipemia in a female subject harboring a loss-of-function *DPP4* mutation with decreased DPP4 activity. Other sporadic *DPP4* mutations verified the low-frequent occurrence of genetic inhibition of DPP4 activity, at least in the Chinese population studied. These results may provide new evidence for evaluation of the potential long-term effects of DPP4i and GLP-1 analogs.

## Introduction

The term incretin refers to all gastrointestinal tract-derived metabolic hormones that regulate postprandial glucose. Glucagon-like peptide-1 (GLP-1) and glucose-dependent insulinotropic polypeptide (GIP) are two well-known incretins with proven effects in promoting insulin secretion and regulating postprandial glucose levels. Previous findings have demonstrated that postprandial GLP-1 regulates glucose more potently than does GIP in its diverse target organs, including pancreatic α and β cells and the central neural (1) and cardiovascular systems (2).

Dipeptidyl peptidase IV (DPP4), a serine exopeptidase that inactivates GLP-1, GIP, and several other peptides by cleaving alanine or proline at the penultimate position from the N-terminus, ubiquitously exists in both membrane-bound form on epithelial cells, fibroblasts, and leukocyte subsets and in soluble form in the circulation (3). Due to rapid degradation by DPP4 (within 2 min), less than 25% of the total amount of GLP-1 secreted remains intact (4). Even for the intact form of circulating GLP-1, 40–50% is degraded in the liver (5). Therefore, only 10–15% of total GLP-1 is stably intact and active [active glucagon-like peptide-1 (AGLP-1)] in the circulation, which reveals a substantial potential for increasing GLP-1 levels by inhibiting DPP4 activity. Accordingly, previous data showed that DPP4 inhibitors (DPP4i), namely, different gliptins (which effectively lowered enzymatic activity by 70–90%), significantly increased the circulating AGLP-1 levels. Considering their glucoregulatory effects by potentiating GLP-1 activity, DPP4i have been regarded as promising anti-diabetic agents with a low incidence of hypoglycemia and weight gain (3). Previous observations showed that DPP4i treatment (12–48 weeks) significantly protected pancreatic β cells in subjects with type 2 diabetes (T2DM) or pre-diabetes (6). After approval by the U.S. Food and Drug Administration (FDA) in 2006, DPP4i use in the clinical treatment of T2DM has dramatically increased over the past decade (from 0.5% in 2006 to 26.4% in 2016). Because of the relative short-term clinical usage of DPP4i, it is of great importance to identify genetic factors affecting human DPP4 activity and to clarify the potential long-term physiological effects of extensive DPP4i use.

In this study, we first identified a 39-year-old female proband showing 25-fold higher plasma AGLP-1 levels than matched controls. Whole-exome sequencing (WES) revealed a novel *DPP4* mutation, p.V486M (c.1456 G>A), in the proband. Further functional studies demonstrated the decreased enzymatic activity of p.V486M mutant and the corresponding higher AGLP-1 level and insulin-promoting effects. In addition to p.V486M, enzymatic activities of another eight sporadic mutations in the *DPP4* gene from our in-home database were identified. Our study represents the first case of an inactivating human DPP4 mutant that could mimic the pharmacological inhibition of DPP4. The current findings may also provide clinical insights into the long-term physiologic and pathophysiologic effects of DPP4i.

## Materials and Methods

### Study Participants

Previously, 240 subjects, including 120 with normal glucose tolerance (NGT), 41 with impaired glucose tolerance (IGT), 9 with type-1 diabetes (T1DM), and 70 with T2DM, were evaluated for postprandial insulin, C-peptide levels, and incretin responses to two carbohydrates [75 g oral glucose (a monosaccharide) and 100 g standard noodles (a polysaccharide) contacting 75 g carbohydrates equivalently] (7). The proband with an unusually high AGLP-1 level, and the parents were further recruited in our study. The oral glucose tolerance test (OGTT) was performed on these participants in the morning after fasting for 10–12 h and no smoking. Before blood drawing, 10 µl DPP4i (Millipore) per 1 ml blood was added to vacuum tubes containing dipotassium ethylenediaminetetraacetic acid for AGLP-1 measurements. Then, blood was drawn into the vacuum tubes in the fasting state and at 30, 60, 120, and 180 min after the 75-g oral glucose load. Blood samples were centrifuged at 3,000 rpm for 15 min to collect serum and plasma samples to measure glucose (Beckman CX-7 Biochemical Autoanalyzer), insulin and C-peptide (Roche Diagnostics), and AGLP-1 [Glucagon Like Peptide-1 (active) ELISA Kit, Millipore] levels. The glucagon and peptide YY (PYY) levels of the study subjects were determined by Milliplex Human Metabolic Hormone Magnetic Panel (HMHMAG-34K) using a Luminex Magpix analyzer (Luminex). Data were analyzed using Milliplex Analyst.V5.1 (Luminex). Clinical information was obtained by performing detailed and comprehensive investigations. In this study, we performed WES for genomic DNA (gDNA) from the trio members. Sanger sequencing (SS) was performed to decode the *DPP4* gene of the proband’s aunt and cousin. We also re-evaluated 10 subjects with 1 of the other 8 missense mutations in the *DPP4* gene that were identified in our in-home WES project, referred to as the “Genetics of obesity in Chinese Youngs (GOCY)” study, which was previously established in the Ruijin Hospital and registered in ClinicalTrials.gov^1^ (identifier NCT01084967) (8). Our study protocol was approved by the Institutional Review Board of Ruijin Hospital, and informed consent was obtained from all study participants. The study conformed to the principles of the Declaration of Helsinki.

### Plasma DPP4 Activity and Serum DPP4 Concentration Assays

Plasma DPP4 activity was measured using the DPP4 Activity Assay Kit (Sigma-Aldrich), according to the manufacturer’s instructions. In this assay, DPP4 cleaves the N-terminal glycine-proline residue of non-fluorescent glycine-proline-7-amido-4-methylcoumarin hydrobromide (Gly-Pro-AMC) and releases fluorescent AMC. The fluorescence was determined every 5 min using a spectrofluorometer (excitation 360 nm/emission 460 nm), until the value of the most active sample exceeded the linear range of the standard curve. The standard curve was generated using 0–1 µmol/l AMC solutions. Serum DPP4 concentrations were measured using the Human CD26 ELISA Kit (RayBiotech).

### Exome Capture and Sequencing

All gDNA extractions from peripheral blood samples of the trio family members were performed using the QIAamp DNA Blood Midi Kit (Qiagen). DNA concentrations were measured using NanoDrop 2000 (Thermo Fisher Scientific), and sheared with Covaris S220 Sonicator (Covaris) to a targeted average length of 300–400 bp. Fragmented DNA was purified using Sample Purification Beads (Illumina). Adapter-ligated libraries were prepared with the TruSeq Nano DNA Sample Prep Kit (Illumina) according to the manufacturer’s protocol. DNA concentrations of the resulting sequencing libraries were measured using Qubit 2.0 Fluorometer dsDNA HS Assay (Thermo Fisher Scientific). Quantities and sizes of the resulting sequencing libraries were analyzed using an Agilent BioAnalyzer 2100 (Agilent). The libraries were used to study cluster formation on an Illumina cBOT cluster-generation system with HiSeq X HD PE Cluster Kits (Illumina). Paired-end sequencing was performed using an Illumina HiSeq X instrument, following Illumina-provided protocols for 2 × 150 paired-end sequencing.

### Variant Analysis

The sequencing reads were aligned with default parameters to the human reference genome sequence build 37 (hg19) using BWA software, version 0.5.8. SamTools was used to convert alignments in SAM format to sorted, indexed BAM files. Both invalid alignments and marked duplicate reads were removed from the BAM files using the Picard tool.^2^ The realignment of BAM files was performed using the GATK RealignerTargetCreator and IndelRealigner tools. The GATK IndelGenotyperV2 and UnifiedGenotyper tools were used to call genotypes following the recommendations from GATK.^3^

### *In Silico* Prediction of Mutation Effects

The potential effects of missense variants were evaluated using SIFT^4^ and Polyphen2.^5^ Conservation of all missense and indels variants in vertebrates was assessed by PhastCons.^6^ SWISS-MODEL^7^ and I-TASSER^8^ analyses were performed to predict the wild-type (WT) and mutant human DPP4 structures, which were further analyzed in PyMOL.^9^

### The Incretin Effect *In Vivo*

The incretin effect test was performed *in vivo* on the morning after overnight fasting (10–12 h) and no smoking. Blood was drawn into vacuum tubes in the fasting state and at 15, 30, 45, 60, 75, 90, 105, 120, 180, and 240 min post-consumption of 75-g oral glucose for glucose, insulin, and C-peptide measurements. After a 7-day washout period, a high concentration of glucose (20%) was administrated intravenously to match the glycemic excursions after oral glucose ingestion. The infusion of glucose was appropriately adjusted according to glucose levels measured every 5 min. Adjustments in the glucose administration rate were recorded at each time point to perform glucose consumption calculations. Similar to the procedure used in the oral glucose load test, blood was drawn into vacuum tubes in the fasting state and at 15, 30, 45, 60, 75, 90, 105, 120, 180, and 240 min during intravenous glucose loading for insulin and C-peptide measurements. The incretin effect was calculated based on the ratio of the difference in the plasma insulin or C-peptide level observed after oral glucose or isoglycemic intravenous glucose administration to the total β cell responsiveness to oral glucose.

### Rat Islet Isolation and Treatment Design

Islets were isolated from the pancreases of male Sprague-Dawley rats by *in situ* collagenase infusion and separated by density gradient centrifugation, as described previously (9, 10). Islets were cultured for 4 h in RPMI 1640 medium containing 5.6 mM glucose and 0.25% bovine serum albumin at 37°C and 5% CO_2_. After pre-incubation, intact rat islets were transferred to 24-well plates (10 islets per well) and cultured in RPMI 1640 medium containing 5.6 mM glucose and other supplements (100 pmol/l exendin-4 or 25% germ-free serum) for a further 24 h at 37°C and 5% CO_2_. Germ-free serum samples of the proband and healthy controls were obtained by using Millex Syring Filter Unit (Millipore), and the former sample was then diluted 5-, 20-, and 100-fold to generate an AGLP-1 level gradient. The islet culture supernatants were collected at 24 h post-treatment to assay for GLP-1-stimulated insulin secretion and insulin contents. Insulin levels were measured using the Rat Insulin ELISA Kit (Alpco), according to the manufacturer’s instructions. In preparation for cytoplasmic insulin content measurement and subsequent normalization, islets were lysed overnight with acidified alcohol consisting of 75% absolute ethyl alcohol, 1.5% hydrochloric acid, and 23.5% tri-distilled water at −80°C.

### Overexpression of WT and Mutant DPP4

WT and mutant human *DPP4* were cloned into the eukaryotic expression vector pEGFP-N1 and overexpressed in human embryonic kidney 293T (HEK293T) cells, which were cultured in Dulbecco’s modified Eagle’s medium (Life Technologies) containing 10% fetal bovine serum at 37°C. Transfections were performed using Lipofectamine 2000 (Invitrogen) at 80–90% confluency. After 48 h, transfectants positive for DPP4 and the maker green fluorescent protein (GFP) were harvested for real-time quantitative polymerase chain reaction (qPCR), western blotting, and DPP4 activity analyses.

### qPCR Experiments

Total mRNA was extracted from HEK293T transfectants and reverse transcribed to cDNA, using a Reverse Transcription System Kit (Promega). Human DPP4 expression were detected by qPCR using specific primers: 5′-GGTTCTGCTGAACAAAGGCA-3′ and 5′-TCTCCAAGAAAACTGAGCTGT-3′. qPCR was performed using SYBR Premix EX Taq (Takara), the Applied Biosystems 7500 qPCR system, and the following thermocycling profile: initial denaturation at 95°C for 10 s followed by 40 cycles of amplification (denaturation at 95°C for 10 s, annealing, and extension at 60°C for 30 s). We normalized the expression levels of WT and mutant *DPP4* mRNA to that of human *36B4* mRNA.

### Western Blot Analysis

Transfected cells were washed twice with phosphate-buffered saline, treated with RIPA/5% NP40 buffer (Caibo) containing 1% protease inhibitor (Protease and Phosphatase Inhibitor Cocktail; Thermo Fisher Scientific), and centrifuged (12,000 × *g*, 4°C, 30 min). Supernatants containing protein fractions were collected, and protein concentrations were determined using the BCA Protein Assay Kit (Thermo Fisher Scientific). Denatured proteins (99°C, 10 min) were separated on 10% resolving gels and 5% stacking gels, and electrotransferred onto polyvinylidene fluoride (PVDF) membranes (Millipore). The membranes were blocked with 5% non-fat powdered milk (BBI Life Science) diluted in TBS-T buffer (Promoton) for 2 h and then incubated overnight in 5% non-fat powdered milk containing anti-GFP (1:1,000, Cell Signaling Technology) at 4°C. After incubation with the primary antibody, the PVDF membranes were incubated for 2 h with a secondary anti-rabbit IgG (1:1,000, Cell Signaling Technology) antibody at room temperature (20–25°C). To detect DPP4, the membranes were incubated with the Immobilon Western HRP Substrate (Millipore), and proteins were visualized using a luminescent image analyzer (ImageQuant LAS 4000; GE Healthcare Life Sciences).

### DPP4 Activity Assay of All Mutants

Transfected cells were harvested, homogenized in a fourfold volume of ice-cold DPP4 assay buffer (Sigma-Aldrich), and completely disrupted using a BioRuptor UCD-200 (Diagenode) at 160 W (30 s on/30 s off for 5 min). Samples were centrifuged at 12,000 × *g* for 15 min to remove insoluble materials. Protein concentrations were measured using the BCA Protein Assay Kit (Thermo). DPP4 activity was determined by measuring the Gly-Pro-AMC hydrolysis rate (Sigma). The reaction was initiated by adding 45 µl sample to 5 µl reaction substrate. The N-terminal Gly-Pro residues were removed from the non-fluorescent substrate by DPP4 to release the hydrolyzed fluorescent product, AMC. The fluorescence was captured using an excitation wavelength of 355 nm and an emission wavelength of 460 nm every 10 s for 5 min, with an Envision microplate reader. The standard curve was generated using 0–100 µM AMC solutions (Sigma-Aldrich).

### Contrast-Enhanced Computed Tomography (CT)

Contrast-enhanced CT was performed with a 16-detector row GE-Lightspeed CT scanner (GE Healthcare). The study subject was placed in the supine position and was injected with 100–150 ml iohexol (350 mg I/ml). The scanning was carried out ranging from diaphragm to the lower edge of horizontal part of duodenum. After unenhanced imaging, dynamic triple-phase contrast enhancement was applied to the subject. After 120-ml contrast medium injection at a flow rate of 3–4 ml/s, the CT acquisitions were obtained at 20–25 s (the arterial phase) and 55–60 s (the portal phase). Images in arterial and portal phases were reconstructed into 2.5-mm slices, and the reconstruction interval was 1.25 mm.

### Anal-Route Double-Balloon Enteroscopy

A clear liquid diet was recommended to the proband for a day before performing enteroscopy. Meanwhile, for bowel preparation, the proband was asked to receive 50-g oral magnesium sulfate at 8:00 p.m. and drink 1,000 ml water. Anal-route double-balloon enteroscopy was performed using an EN-450T5 (FUJIFILM Medical) as describe previously (10). During the endoscopic procedure, the apparatus was eventually advanced to the distal ileum. At the end of the advancement period, the sonde of enteroscope was slowly withdrew, and the biopsy specimens of ileum and colon tissues were obtained.

### Hematoxylin/Eosin (HE) and Immunohistochemical Staining

The paraffin-embedded sections of ileum and colon tissue were prepared and processed for HE as described previously (11). The ileum and colon tissues pathology of the proband in HE was examined by light microscope (LM) (H500S, Nikon). Immunohistochemical staining of the paraffin-embedded sections was performed according to EnVision system protocol. The sections were incubated with anti-Chromogranin A (1:200, Abcam) and with HRP-labeled secondary antibody (DAKO). The localization and distribution of intestinal endocrine cells were examined under the H500S LM, and the images were processed by Image-pro Plus software, version 9.3 (Media Cybernetics).

### Transmission Electron Microscopy (TEM)

The biopsy samples were sectioned into 1 mm^3^ with a cold blade and fixed with 4% glutaraldehyde. After dehydration by a graded series of ethanol, the specimen was embedded in embedding medium and processed to ultrathin sections. The sections were then stained by uranyl acetate and alkaline lead citrate. Finally, the ultrastructure of the ileum and colon tissues were observed with a TEM (CM-120, Philips).

### Statistical Analysis

All statistical analyses were performed using SAS software, version 9.3 (SAS Institute). Analysis of variance (single-factor variation) was used to determine the effects of different *DPP4* mutations on expression levels and enzymatic activities. Correlations between DPP4 activity and clinical phenotypes, including glucose and lipids parameters, were tested by determining Pearson’s correlation coefficient. The data are presented as the mean and SEM, or the mean and the 95% confidence interval, and *P* < 0.05 was considered statistically significant. In addition, ImageJ software (version 1.49) was used to quantify protein band densities in western blot films. Bands were quantified as the relative density of a protein band of interest to that of the loading control.

## Results

### A Proband with Hyperglipemia (Hyper-Glucagon-Like Peptide-1-Emia)

**Figure 1:**
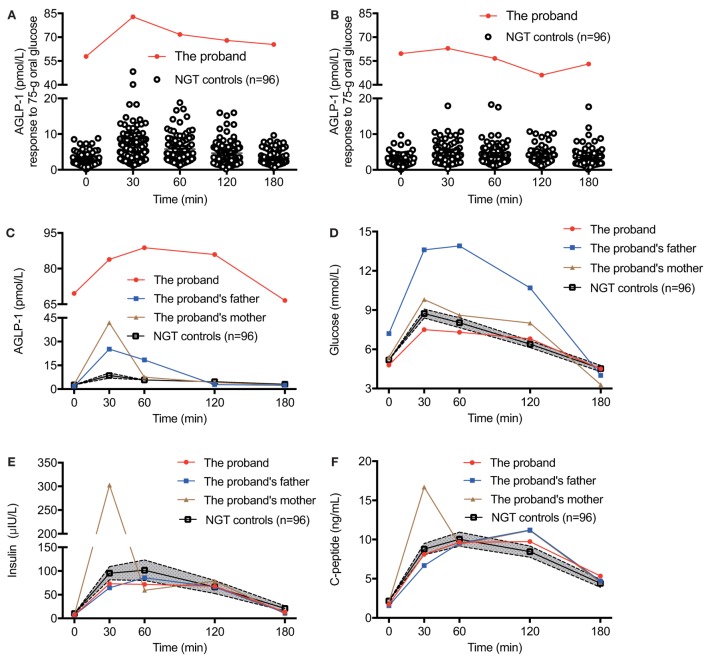
The glycometabolism of the proband with elevated plasma AGLP-1 level. The plasma AGLP-1 level of the proband measured in the fasting state (0 min) and at 30, 60, 120, and 180 min after consumption of 75-g oral glucose **(A)** or 100-g standard noodle **(B)**. The plasma AGLP-1 **(C)**, plasma glucose **(D)**, serum insulin **(E)**, and C-peptide **(F)** levels of the trio family and matched controls (*n* = 96) were measured in the fasting state (0 min) and at 30, 60, 120, and 180 min post-consumption of 75-g oral glucose. Data were expressed as the mean and the 95% confidence interval. AGLP-1, active glucagon-like peptide-1; NGT, normal glucose tolerance.

We hypothesized that individuals with impaired DPP4 activity could be clinically characterized with significantly increased AGLP-1 levels. Thus, we screened 240 participants with NGT, IGT, T1DM, or T2DM whose AGLP-1 levels were measured previously (7). Of note, we found a 39-year-old female with an unusually elevated AGLP-1 level in the fasting state (69.6 pmol/l), approximately 25-fold higher than the mean value of other 96 NGT participants, which we characterized as “hyperglipemia.” AGLP-1 level was also significantly higher in response to a 75-g oral glucose load at different time points (83.9 pmol/l at 30 min, 88.8 pmol/l at 60 min, 86.0 pmol/l at 120 min, and 66.6 pmol/l at 180 min) compared with other subjects (Figure 1A). Similar results were obtained in respond to 100-g standard noodles load (Figure 1B). The clinical values of postprandial glucose, insulin, C-peptide, GLP-1, and glucagon levels of the proband to these two carbohydrates were similar, and glucagon levels were within the normal range (25–250 ng/l) (Table S1 in Supplementary Material). We further recruited the parents of the proband. The plasma AGLP-1 level was higher in the proband than her parents and slightly higher at 30 min in her parents compared with NGT controls (Figure 1C). However, no significant differences in glucose and insulin levels were found during the OGTT between the proband and NGT controls, although glucose level was higher in her father who was diagnosed as T2DM (Figures 1D,E). Regarding the C-peptide, the proband and her father showed a higher level at 120 min post-loading and a delayed decline (Figure 1F). In addition, glucagon levels of the parents of the proband in the fasting state and at 30, 60, 120, 180 min post-loading were also within the normal range, and no significant difference between among the trio members in PYY levels (Figure S1 in Supplementary Material).

### The Incretin Effect of Hyperglipemia in the Proband *In Vivo*

**Figure 2:**
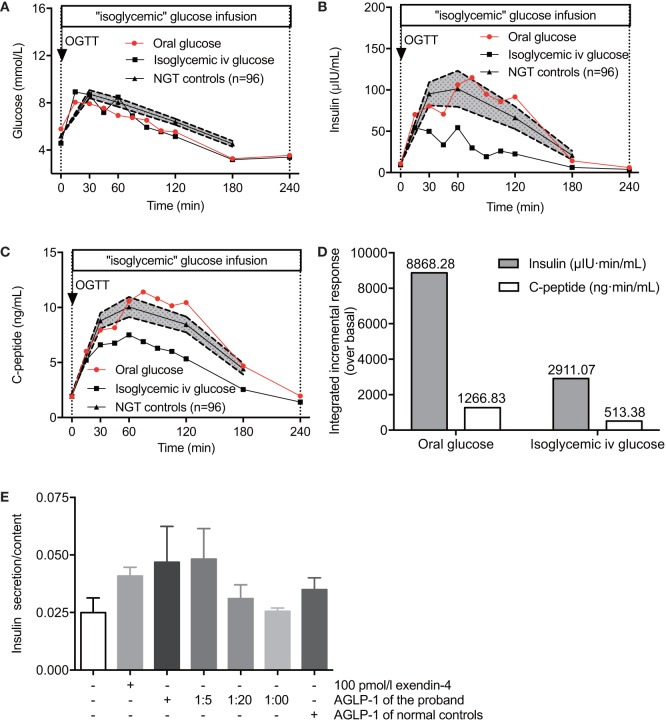
The incretin effect of hyperglipemia *in vivo* and *ex vivo*. The plasma glucose **(A)**, serum insulin **(B)**, and C-peptide **(C)** levels of the proband and NGT controls (*n* = 96) in response to 75-g oral glucose consumption or isoglycemic intravenous glucose administration. **(D)** Comparisons of the insulin and C-peptide responses following 75-g oral glucose consumption and isoglycemic intravenous glucose administration. **(E)** Rat primary islets were treated with a serial AGLP-1 gradient for 24 h and collected for insulin secretion measurements. Data were expressed as mean ± SEM. AGLP-1, active glucagon-like peptide-1; OGTT, oral glucose tolerance test; NGT, normal glucose tolerance.

To evaluate the incretin effect of elevated AGLP-1, we performed 75-g OGTT and isoglycemic intravenous glucose tests in the proband. The total amount of glucose administrated during the isoglycemic intravenous infusion was 31.3 g, which was designed to match glycemic excursion after oral glucose ingestion (Figure 2A). As expected, oral glucose intake induced higher insulin and C-peptide secretory responses compared with isoglycemic intravenous infusion (insulin 8,868.3 vs. 2911.1 μIU·min/ml and C-peptide 1266.8 vs. 513.38 ng·min/ml; Figures 2B–D). Based on the integrated incremental responses (trapezoidal rule) of insulin and C-peptide, the calculated incretin effects for insulin and C-peptide (67.2 and 59.5%, respectively) were actually similar to those of the NGT subjects described previously (12, 13), suggesting there was no elevated incretin effect in according to the increased AGLP-1 level in the proband.

### Hyperglipemia-Stimulated Insulin Secretion Assay *Ex Vivo*

Although no increased incretin effect of gastrointestinal tract was detected *in vivo*, we observed a relatively enhanced acute incretin effect of the serum from the proband in comparison to normal controls in an *ex vivo* study (Figure 2E). β cells in isolated rat islets responded to either 100 pmol/l exendin-4 or high titer levels of the proband’s serum to produce more insulin than control islets without stimulators. Islets cultured with original serum from the hyperglipemic proband showed higher insulin secretion in response to glucose than when cultured with serum from healthy controls (0.047 vs. 0.035, relative values), and the effect was decreasing with high dilution showing a dose-dependent manner (0.047, 0.048, 0.031, and 0.026, respectively). Of note, when the proband’s original serum was diluted 20-fold to reach roughly the same AGLP-1 levels of serum from healthy control subjects, similar incretin effects were observed (0.031 vs. 0.035, relative values). These results suggested that higher AGLP-1 level in the serum from the proband has increased incretin effect to promote insulin release. The degradation pathway of GLP-1 was further examined to explore the potential causes for hyperglipemia.

### A Novel *DPP4* Mutation Identified in the Proband, Her Father, and Aunt

**Figure 3:**
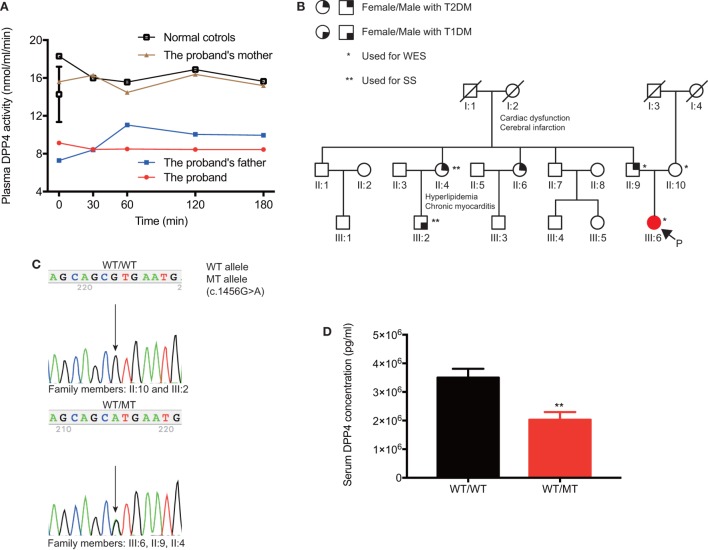
Clinical features and genetic information of *DPP4* in the pedigree. **(A)** The comparison of plasma DPP4 activity between the proband and her parents. **(B)** Family members are designated by Arabic numerals. Horizontal lines between individuals represent marriage. Vertical lines represent lineage. The individual’s current clinical diagnosis and genotypes are provided if the blood samples were available. **(C)** Sanger sequencing was applied to verify genotypes, and representative chromatograms are shown. Individuals who are heterozygous for the p.V486M (c.1456 G>A) mutation showed overlapping A and G peaks. **(D)** Comparison of serum DPP4 concentrations between the mutation carriers and non-mutation controls. Data were expressed as mean ± SEM. **P-value <0.01. DPP4, dipeptidyl peptidase IV; T1DM, type 1 diabetes; T2DM, type 2 diabetes; WES, whole-exome sequencing; SS, sanger sequencing; P, the proband; WT, wild-type; MT, mutant-type.

Secreted GLP-1 was rapidly degraded by DPP4 enzyme. We next performed enzymatic activity measurement to evaluate the plasma DPP4 activity of the proband, her parents, and healthy control subjects. The fasting plasma samples of the proband and her father hydrolyzed Gly-Pro-AMC to yield 9.1 and 7.3 nmol/ml AMC per min at 37°C, which was strikingly lower than that of her mother (15.6 nmol/[ml·min]) and matched controls (12.7 ± 1.1 nmol/[ml·min]) (Figure 3A). The plasma DPP4 activity at 30, 60, 120, and 180 min after oral glucose intake were consistent with the fasting state. Thus, we found that the proband (III:6) and her father (II:9) showed similarly decreased DPP4 activity, which indicated that hyperglipemia in the proband could originate from her father (Figure 3B).

To identify the potential genetic defects in the proband, gDNA from the trio members was analyzed by WES. Among 57,356 variants in an autosomal-dominant mode, 43,593 passed the primary quality control filters, and 43,370 were filtered out as homozygotes, validated polymorphisms in several resources [dbSNP142, 1,000 genomes, NHLBI Exome Sequencing Project Exome Variant Server, and our in-home GOCY WES database (8)], and variants not shared by the proband and her father. Of the remaining 219 variants, 78 variants leading to nonsense, missense, or frameshift mutations, or affecting splice sites were proposed as being potentially deleterious (Table 1). An in-depth and extensive literature review of the remaining variants (Table S2 in Supplementary Material) enabled prioritization of one variant, p.V486M (c. 1456 G>A), a novel mutation in the *DPP4* gene responsible for degrading GLP-1. The heterozygous p.V486M mutation in the *DPP4* gene was validated in the proband and her father by SS, but was not present in her mother or the 448 subjects of the GOCY WES study (Figure 3C). We also employed SS to identify the heterozygous p.V486M mutation in the *DPP4* gene of the proband’s aunt (II:4, 67 years old) with T2DM, hyperlipidemia, and chronic myocarditis. Other members in the pedigree have underwent neither WES nor SS.

**Table 1:** Identifying the gene associated with a high active glucagon-like peptide-1 level in the pedigree by exome sequencing analyses.

Filter	Dominant	Recessive	*De novo* mutations
Passing primary quality control filter	43,593	13	25
Frequency >0.01 in dbSNP142, 1,000 Genomes, and esp6500	1,348	3	14
Not in 221 lean controls	1,334	3	14
An autosomal-dominant mode of inheritance (variants shared with the female and her father)	219	0	0
Exonic + splice sites + indels	130	0	0
Non-synonymous variants/donor site mutations/coding indels	78	0	0

Importantly, we further verified that the serum DPP4 concentrations of all p.V486M carriers (II:4, II:9, and III:6) were significantly lower compared with non-carriers (2.70 ± 0.27 vs. 4.72 ± 0.24 µg/ml, *P* = 0.01) (Figure 3D). These data suggested that the key clinical phenotypes (reduced DPP4 activity and concentration) co-segregated with their *DPP4* variant genotype.

### Functional Assessment of p.V486M and Other Eight Non-Synonymous *DPP4* Variants Identified in our Database

To comprehensively evaluate genetic *DPP4* defects in a population, we analyzed all *DPP4* variants in the coding region in 227 young, obese subjects and 221 lean controls from the GOCY WES study (8). Another eight rare/low-frequency non-synonymous *DPP4* variations, namely, p.D65E (c.195 T>A), p.W124* (c.371 G>A), p.P290L (c.869 C>T), p.A291P (c.871 G>C), p.S323L (c.968 C>T), p.T351A (c.1051 A>G), p.R492K (c.1475 G>A), and p.K554Q (c.1660 A>C), were included for further functional studies (Table 2).

**Table 2:** Mutations in the *DPP4* gene identified in individuals in our study.

Chromosome	Position^a^	Reference^a^	Variant allele	Amino acid change (one-letter code)	dbSNP142	Type of mutation	Functional prediction	
							Polyphen2	SIFT
2	162903515	A	T	p.D65E	Not available	Missense	Benign	Tolerated
2	162895690	C	T	p.W124*	Not available	Nonsense	Not available	Not available
2	162890069	G	A	p.P290L	Not available	Missense	Probably damaging	Damaging
2	162890067	C	G	p.A291P	Not available	Missense	Benign	Tolerated
2	162881369	G	A	p.S323L	Not available	Missense	Probably damaging	Tolerated
2	162879282	T	C	p.T351A	Not available	Missense	Benign	Damaging
2	162873630	C	T	p.V486M	Not available	Missense	Benign	Tolerated
2	162873370	C	T	p.R492K	Not available	Missense	Benign	Tolerated
2	162868475	T	G	p.K554Q	Not available	Missense	Benign	Tolerated

*^a^Mutation numbering is based on NCBI Gene ID 1803, Transcript ID NM_001935, and Protein ID NP_001926.2*.

**Figure 4:**
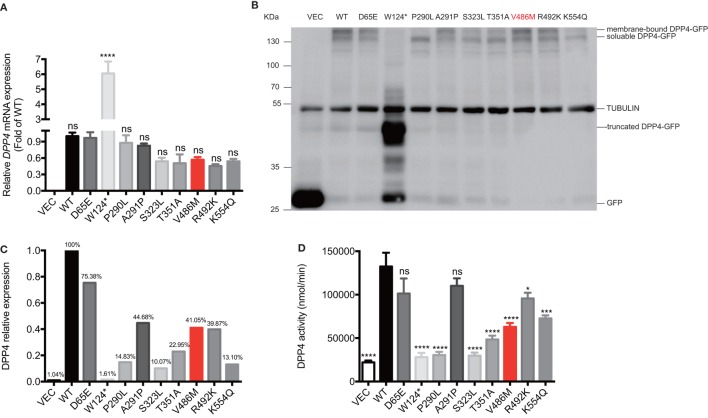
Biological effects of *DPP4* mutations. The mRNA expressions **(A)**, protein expression **(B,C)**, and the enzymatic activities **(D)** of DPP4 mutants and wild-type DPP4 overexpressed in human embryonic kidney 293T cells. Data were expressed as mean ± SEM. *P-value <0.05, ***P-value <0.001, ****P-value <0.0001 vs. WT group. DPP4, dipeptidyl peptidase IV; VEC, vector; WT, wild-type; GFP, green fluorescent protein.

All recombinant plasmids carrying WT or mutant *DPP4* variants were overexpressed in HEK293T cells. Although the p.V486M mutation did not alter the mRNA levels of *DPP4* (Figure 4A), western blotting showed a significant reduction (~59.0%) in protein expression (Figures 4B,C). In addition, the protein expression levels of another six DPP4 mutants (p.W124*, p.P290L, p.S323L, p.T351A, p.R492K, and p.K554Q) were also decreased on average by 82.9%, ranging from 24.6 to 98.4%. We further measured the enzymatic activities of DPP4 mutants that were overexpressed in HEK293T cells, finding that activity of the p.V486M mutant was ~47.7% lower than that of the WT protein (63.00 ± 4.47 vs. 132.44 ± 15.87 μmol/[ml·min], *P* = 0.01). These results together linked the genetic *DPP4* mutation to the clinical hyperglipemia feature. In addition to the p.V486M mutant, another six mutants also showed significantly lower activity (Figure 4D), which also indicated that loss-of function *DPP4* mutations are not rare events (~1.3%), at least in Chinese populations.

### Associations between DPP4 Activities and Clinical and Biochemical Characteristics

We next examined the correlation of DPP4 activities and metabolic parameters in DPP4 mutant carriers, and found DPP4 activities correlated negatively with lipid metabolism in terms of total cholesterol (TC, *r* = −0.77, *P* = 0.02) and low-density lipoprotein cholesterol (LDL-c, *r* = −0.73, *P* = 0.03) (Table 3). However, no significant association of DPP4 activity with glucose metabolism was found, such as the fasting and the postprandial plasma glucose levels, serum insulin levels, homeostatic model assessment (HOMA)-insulin sensitivity, and HOMA-insulin resistance, and HOMA-β, in various *DPP4* mutations carriers from this young cohort (age less than 30 years old).

**Table 3:** Correlations between relative DPP4 activities and clinical and biochemical characteristics.

	Correlation coefficient	*P*-value
Age (years)	−0.06	0.86
Body weight (kg)	0.04	0.90
Body mass index (kg/m^2^)	0.04	0.91
Waist circumference (cm)	0.01	0.97
Plasma glucose level (mmol/l)		
Fasting plasma glucose	−0.04	0.91
Postload 30-min plasma glucose	0.26	0.51
Postload 60-min plasma glucose	0.40	0.33
Postload 120-min plasma glucose	0.05	0.89
Postload 180-min plasma glucose	0.15	0.73
Serum insulin level (μIU/l)		
Fasting serum insulin	0.09	0.80
Postload 30-min serum insulin	−0.55	0.16
Postload 60-min serum insulin	−0.37	0.42
Postload 120-min serum insulin	0.08	0.85
Postload 180-min serum insulin	0.08	0.85
Glycated hemoglobin (%)	−0.22	0.72
HOMA-IS	−0.20	0.56
HOMA-IR	0.01	0.99
HOMA-β	−0.06	0.86
Fasting serum cholesterol (mmol/l)		
Total	−0.77	0.02*
HDL-c	−0.15	0.69
LDL-c	−0.73	0.03*
Fasting serum triglyceride (mmol/l)	0.02	0.96

*HOMA-IS, homeostatic model assessment-insulin sensitivity; HOMA-IR, homeostatic model assessment-insulin resistance; HOMA-β, homeostatic model assessment-β cells; HDL-c, high-density lipoprotein cholesterol; LDL-c, low-density lipoprotein cholesterol; DPP4, dipeptidyl peptidase IV*.

*P < 0.05.

### The 4-Year Follow-up Data of the Proband

During the 4-year follow-up of the proband, her fasting blood glucose and lipids parameters, such as TC and LDL-c levels, have gradually increased (Table 4), suggesting a deterioration of glucose and lipids metabolism. Several serum tumor makers, especially CA125, have increased markedly over the course of 4 years, implying a high risk of developing tumors. Although we performed contrast-enhanced CT (targeting the pancreas) and enteroscopy (targeting the intestines), and analyzed the intestinal biopsy samples, no positive signs, such as tumor or local hyperplasia in these two tissues, were detected at the fourth year of the follow-up period (Figure S2 in Supplementary Material). In addition, there is no significant changes in the body weight or eating/exercise habits in the proband during the follow-up.

**Table 4:** The 4-year follow-up results of glucose and lipids metabolism of the proband.

Time (years)	FBG (mmol/l)	TG (mmol/l)	TC (mmol/l)	HDL-c (mmol/l)	LDL-c (mmol/l)	CA199 (U/ml)	CEA (ng/ml)	CA242 (U/ml)	CA724 (U/ml)	CA15-3 (U/ml)	CA125 (U/ml)
Reference range	3.9–6.1	0.56–1.70	2.33–5.70	0.80–1.80	1.30–4.30	<35.00	<5.00	<20.00	>6.90	<35.00	<35.00
1st	4.78	1.08	3.61	1.27	1.86	45.05	1.35	28.93	1.05	6.83	92.16
2nd	5.05	1.35	3.80	1.31	2.01	26.15	2.14	19.80	2.31	9.76	211.27
3rd	5.61	0.56	4.24	1.49	2.35	62.42	2.85	34.58	2.95	13.73	>500.00
4th	6.02	1.74	4.06	1.41	2.17	21.50	1.57		0.65	23.60	362.00

*FBG, fasting blood glucose; TG, triglyceride; TC, total cholesterol; HDL-c, high-density lipoprotein-cholesterol; LDL-c, low-density lipoprotein-cholesterol*.

## Discussion

It is well established that the dynamic balance between biosynthesis and degradation maintains a stable AGLP-1 level in human circulation (1). Any disturbances in the balance, such as increased production and/or decreased degradation, are theoretically expected to generate elevated AGLP-1 levels followed by a GLP-1-insulin-glucose homeostasis imbalance. DPP4i, an antidiabetic drug which functions to increase circulating AGLP-1 by inhibiting DPP4 activity, has been widely used for the treatment of T2DM (14). In 2016, DPP4i is marketed 107.6 billion dollars globally as the second best-selling anti-diabetic drugs. However, concerns also raised as DPP4i usage has shown increased risk of hospitalization for pancreatitis and pancreatic tumors in patients with diabetes (15–17). In addition, the hypoglycemic DPP4i, including saxagliptin and sitagliptin, were reported to promote existing tumor metastasis by enhancing cancer cell mobility and invasive capacity in an animal study (18).

Considering the relatively short-term clinical use of GLP-1 mimetic drugs (FDA-approved in 2005) and DPP4i (FDA-approved in 2006) with diabetic patients, it is hard to determine the long-term adverse effects of incretin therapy over several decades. Although ablation of DPP4 activity has been extensively evaluated in both rat and mouse models, subjects carrying *DPP4* mutations with impaired DPP4 activity, which are critical for evaluation of long-term physiological effects of GLP-1, yet have not been reported. In this study, we performed clinical tests, genetic analysis and validation, and molecular biology experiments to explore the novel loss-of-function p.V486M (c.1456 G>A) mutation in *DPP4*, which potentially accounts for the extremely high AGLP-1 levels in the proband, and further assessed subsequent glucose and lipid metabolism, as well as tumor markers.

We first identified the hyperglipemia phenotype of the proband showing higher AGLP-1 levels than controls. Regarding the reasons for the increased AGLP-1 levels, a specific L cell tumor with high GLP-1 secretion was excluded due to negative examination in contrast-enhanced CT, enteroscopy, and LM and TEM of intestinal tissues (Figure S2 in Supplementary Material). Furthermore, no functional mutations in the *GLP1R* and its downstream genes were identified either. Thus, we further focused on DPP4, the enzyme degrading GLP-1. By using WES, we identified p.V486M mutation in the *DPP4* gene in the proband who correspondingly showed reduced activity and serum concentration of DPP4 than non-mutation carriers. It was a high possibility that this *DPP4* defect accounted for the higher AGLP-1 level (hyperglipemia) in the proband. Other potential factors that may also contribute to clinical hyperglipemia features could not be thoroughly excluded at present.

Several important findings need further clarification. First is the apparent discrepancy of plasma AGLP-1 levels between the proband and her father, both of whom carried the DPP4 p.V486M mutation and showed lowered plasma DPP4 activity while her father did not show comparably high AGLP-1 level. The proband’s father was 69 years old at the time of publication and was diagnosed with T2DM at 59 years of age. Patients with T2DM displayed significantly reduced L-cell function and plasma AGLP-1 levels, particularly in the late phase after oral glucose consumption (13). Unfortunately, we could not acquire the father’s blood samples to measure his AGLP-1 level at 39 years of age (the same age of the proband at present). However, we postulate that the AGLP-1 levels of the father might be masked and declined gradually to a normal range with the development of T2DM. Assessing AGLP-1 levels in the *DPP4^−^*^/^*^−^* mice at different ages, especially at older ages, would be helpful to examine this question. Of note, we did not observe hypoglycemia in the proband despite the increased AGLP-1 levels, which is different from rare patients with GLP-1 and glucagon co-secreting pancreatic neuroendocrine tumor who displayed hyperinsulinemic hypoglycemia and the proliferation of β-cell (19, 20). This discrepancy might be due to the relatively long exposure time of AGLP-1 levels in the proband, which might lead to some compensations to maintain the glucose balance, while the relatively short exposure time of AGLP-1 levels in the tumor patients did not. Besides, the phenotype of *DPP4^−/−^* mice with high GLP-1 level at born but showed normal fasting blood glucose levels (21) and clinical phenomenon of T2DM patients with GLP-1 analogs but high glucose levels could also partly explain the clinical characterizations of p.V486M carriers.

Second, whether the proband would develop T2DM in the future as her father did still need long-term follow-up, as her father and aunt got T2DM albeit with suppressed DPP4 activity. These puzzles may be partially explained from the findings in a humanized mouse model showing GLP-1-induced short-term glucose-lowering benefits, but long-term damages to β cell functions (22). These long-term deleterious effects could result from GLP-1-induced insulin secretion in β cells and hyperinsulinemia, thereby leading to decreased insulin sensitivity and/or augmented priming effects on β cells (22, 23). Additionally, DPP4 is also known as CD26, a highly glycosylated transmembrane protein expressed on T cells. The T cell-surface molecule CD26/DPP4 plays a crucial role in CD4^+^ T cell development, maturation, and migration. *DPP4^−/−^* mice displayed a reduction in the percentage of CD4^+^ T cells, lowered IL-4 levels, and increased IL-10 levels (24). DPP4 regulated immune responses by decreasing inflammation *via* the action of transforming growth factor-β1 (25). In addition, DPP4 was negatively associated with inflammation in terms of proinflammatory chemokines such as stromal cell-derived factor-1, which is involved in human and experimental arthritis (26). Besides of AGLP-1, DPP4 could also regulate the plasma levels of many other hormones, including GIP, glucagon-like peptide-2, PYY, neuropeptide Y, diazepam-binding inhibitor, meprin-β, sorbitol dehydrogenase, elongation factor-1α, and elongation factor-2, indicating the essential role of DPP4 in peptide and glucose metabolism in a non-GLP-1-dependent manner (27). Genetic or pharmaceutic reduction of DPP4 activity can lead to potential perturbations in immunity, inflammation, and active peptide hormone activity; thus, the long-term outcome of its long-term inhibition should be systemically evaluated, in addition to the effects of elevated AGLP-1 levels. Importantly, glucagon-expressing neuroendocrine tumors as well as microadenomas were present in the pancreases of T2DM donors who have been treated with sitagliptin or exenatide for 1 year or more, implying the possible long-term deleterious potentiation of incretion in developing neuroendocrine tumors (28). According to the baseline and the follow-up data, the proband with the p.V486M mutation and subsequently impaired DPP4 activity seemed to be clinical symptom-free thus far, which indicated that long-term inhibition of DPP4 activity (42 years) was relatively safe. However, of note, the steady deterioration of glucose and lipid metabolism and the markedly increase of serum tumor markers in the proband still raised concerns on the safety and long-lasting effectiveness of hypoglycemic DPP4i to treat diabetes.

Third, we noticed the marked discordance between the clinical and biochemical observations and the *ex vivo* insulin secretion experiments in rat islets. While the observation of normal insulin and C-peptide levels of the proband in our study and diabetic patients described in previous studies (29) may suggest that the incretin effect of long-term hyperglipemia *in vivo* might lead to “GLP-1 resistance,” reduced β cell insensitivity to GLP-1, or could be compensated by some unknown factors *in vivo*. These findings are consistent with the phenotype of *DPP4*^**−/−**^ mice, which showed normal blood glucose and insulin levels under the fasting state (21). Based on our follow-up data of the proband, the steady deterioration of glucose and lipid metabolism also indicated the potential damage of insulin secretion. And a recent study has claimed that long-term GLP-1 use could lead to a reduction in insulin secretion mediated by increasing acetylcholine secretion from α-cell (22, 23). Thus, the direct and indirect effects of the *DPP4* mutation on other tissues and cells, such as immune, endocrine, and nervous system, might contribute to *in vivo* and *ex vivo* discordance. However, further studies will be needed to determine whether these effects represent plausible explanations for the discordance.

Fourth, based on our findings, six additional mutations (p.W124*, p.P290L, p.S323L, p.T351A, p.R492K, and p.K554Q) in the *DPP4* also resulted in reduced DPP4 expression and activities. The genetic inhibition of DPP4 by missense mutations is not a rare event in the general population, at least for Chinese people, which is of a great clinical significance in promoting personalized medicine for carriers of *DPP4* mutations, especially before beginning incretin therapy with DPP4i and GLP-1 mimetic drugs. Thus far, only a few reports have described associations between some single-nucleotide polymorphisms of the *DPP4* gene and dyslipidemia, including, triglyceride, TC, HDL-c, and LDL-c levels (30, 31). Consistently, in our study, decreased enzymatic activity induced by *DPP4* mutations was associated with increased TC and LDL-c levels. These findings were further supported by the follow-up lipid changes of the proband found in a 4-year follow-up.

Some limitations of the study, however, still need careful consideration. One limitation was the lack of detailed clinical and genetic information for other diabetic members in the pedigree of the proband, due to the lack of available blood samples. In this pedigree, we observed that the father and the aunt, the p.V486M mutation carriers, were diagnosed as T2DM. The aunt also had hyperlipidemia and myocarditis, and the grandmother who was postulated as the origin of the p.V486M mutation had a stroke at her old age. A long-term follow-up study of the proband is essential for providing deep insights into the potential effects of long-term reduced DPP4 activity and subsequent high AGLP-1 levels on the function of α cells, β cells, L cells, CD4^+^ T cells, and the cardiovascular system (24, 28, 32). Previous data showed that long-term DPP4 inhibition promoted cardiac fibrosis, inflammation, and reduced ventricular function in older diabetic mice (32). Further mechanistic studies on the role of DPP4 in GLP-1-insulin-glucose homeostasis and cardiometabolic outcomes should be performed with elderly *DPP4*-knockout mice or even with monkey models. Several other functionally damaged DPP4 mutants/variants were identified in a relatively small-scale Chinese population. However, due to lack of AGLP-1 measurement in these subjects, we currently could not determine the effects of these mutations on AGLP-1 levels. Screening of more *DPP4* mutations and exploring their relationship with AGLP-1 levels and T2DM will further clarify the effects of this signaling in glucose homeostasis.

In conclusion, we provide the first report that an adult female with hyperglipemia had inherited a loss-of-function mutation from her father in the *DPP4* gene, which effectively blunted its enzymatic activity in the blood and showed a role in dominating the AGLP-1 levels. Carriers of *DPP4* mutations are considered appropriate and valuable indicators of long-term cardiometabolic and tumor outcomes of DPP4i and incretin mimetic drugs during T2DM treatment. It might also be helpful to evaluate the genetic mutation in the *DPP4* gene before initiating clinical applications with these drugs.

## Ethics Statement

Our study protocol was approved by the Institutional Review Board of Ruijin Hospital, and informed consent was obtained from all study participants. The study conformed to the principles of the Declaration of Helsinki.

## Author Contributions

JW and YZ contributed to the study conception and design. DZ contributed to the implementation of the study and the methods, statistical analysis, results and conclusions section. SZ, XW, MS, WL, QM, and JL contributed to the implementation of the methods. RL, WG, and JH contributed to the analysis and interpretation of data. DZ drafted the work, and JW, RL, and YZ revised it critically for important intellectual content. GN, JW, and YZ supervised the study. All authors read and gave final approval of the version to be published. All authors agreed on the order in which their names will be listed in the manuscript.

## Conflict of Interest Statement

The authors declare that the research was conducted in the absence of any commercial or financial relationships that could be construed as a potential conflict of interest.

## Footnotes

^1^http://clinicaltrials.gov/.

^2^http://sourceforge.net/projects/picard/.

^3^https://software.broadinstitute.org/gatk/best-practices/.

^4^http://sift.jcvi.org/.

^5^http://genetics.bwh.harvard.edu/pph2/.

^6^http://compgen.cshl.edu/phast/.

^7^https://swissmodel.expasy.org/.

^8^http://zhanglab.ccmb.med.umich.edu/I-TASSER/.

^9^http://www.pymol.org/.

## Supplementary Material

Figure S1 The glycometabolism of the proband and her parents. The plasma GIP **(A)**, glucagon **(B)**, PYY **(C)** and TNF-α **(D)** levels of the proband and her parents measured in the fasting state. GIP, glucose-dependent insulinotropic polypeptide; PYY, peptide YY; TNF-α, tumor necrosis factor-α.

Figure S2 Results of image examinations and molecular biology technology methods of the proband at the fourth year of the follow-up. **(A)** In contrast-enhanced computed tomography, the pancreas with complete and smooth envelope had normal size and density, and showed homogenous enhancement. **(B)** The anal-route double-balloon enteroscopy observed no mucous membrane pathological change situation in the ileum and colon. **(C,D)** The morphology of the intestinal mucosal cells in the ileum and colon was normal from the light microscopical appearances (200 X). **(E,F)** Immunohistochemical staining of chromogranin A localized intestinal endocrine cells in ileum and colon tissues under the light microscopy (200 X). **(G,H)** Transmission electron microscopy observed the ultrastructure of intestinal endocrine cells in ileum (7400 X) and colon tissues (4200 X).
