# Supplementary material for: Clinical and Physiological Characterization of Elevated Plasma Glucagon-Like Peptide-1 Levels (Hyperglipemia) in a Dipeptidyl Peptidase IV Mutation Carrier
Source: Front Endocrinol (Lausanne). 2018 Mar 5;9:62. doi: 10.3389/fendo.2018.00062 (PMC5845420; doi:10.3389/fendo.2018.00062)
Supplement: Supplementary file 3 [file Table_1.DOCX]

**Supplementary Table 1.** Postprandial insulin, C-peptide, and incretin responses of the proband to two carbohydrates

|  | 75-g oral glucose | 100-g standard noodles |
| --- | --- | --- |
| Plasma glucose level (mmol/L) |  |  |
| Fasting plasma glucose | 4.80 | 5.11 |
| Postload 30-min plasma glucose | 7.50 | 8.77 |
| Postload 60-min plasma glucose | 7.30 | 8.09 |
| Postload 120-min plasma glucose | 6.80 | 6.49 |
| Postload 180-min plasma glucose | 4.50 | 5.93 |
| Serum insulin level (µIU/L) |  |  |
| Fasting serum insulin | 6.94 | 7.14 |
| Postload 30-min serum insulin | 73.16 | 80.82 |
| Postload 60-min serum insulin | 71.83 | 96.47 |
| Postload 120-min serum insulin | 67.85 | 48.25 |
| Postload 180-min serum insulin | 13.62 | 31.32 |
| Serum C-peptide level (ng/mL) |  |  |
| Fasting serum C-peptide | 1.72 | 1.96 |
| Postload 30-min serum C-peptide | 8.10 | 8.31 |
| Postload 60-min serum C-peptide | 9.64 | 10.29 |
| Postload 120-min serum C-peptide | 9.74 | 9.23 |
| Postload 180-min serum C-peptide | 5.34 | 7.01 |
| Plasma AGLP-1 level (mmol/L) |  |  |
| Fasting plasma AGLP-1 | 57.83 | 59.69 |
| Postload 30-min plasma AGLP-1 | 82.84 | 62.98 |
| Postload 60-min plasma AGLP-1 | 71.73 | 56.65 |
| Postload 120-min plasma AGLP-1 | 67.95 | 46.09 |
| Postload 180-min plasma AGLP-1 | 65.39 | 53.11 |
| Plasma glucagon level (pg/mL) |  |  |
| Fasting plasma glucagon | 97.67 | 84.33 |
| Postload 30-min plasma insulin | 80.81 | 64.62 |
| Postload 60-min plasma insulin | 90.47 | 63.83 |
| Postload 120-min plasma insulin | 73.64 | 54.18 |
| Postload 180-min plasma insulin | 77.30 | 53.55 |
