# Supplementary material for: Clinical and Physiological Characterization of Elevated Plasma Glucagon-Like Peptide-1 Levels (Hyperglipemia) in a Dipeptidyl Peptidase IV Mutation Carrier
Source: Front Endocrinol (Lausanne). 2018 Mar 5;9:62. doi: 10.3389/fendo.2018.00062 (PMC5845420; doi:10.3389/fendo.2018.00062)
Supplement: Supplementary file 4 [file Table_2.DOCX]

**Supplementary Table 2.** Overview of 78 candidate variants revealed in whole-exome sequencing.

| Inheritance | Gene | Chr | Position | Ref | Var | Type of mutation | Polyphen2 | SIFT |
| --- | --- | --- | --- | --- | --- | --- | --- | --- |
| Paternal | NPHP4 | chr1 | 5947457 | CGTATTCA | C | Fs del | NA | NA |
| Paternal | THAP3 | chr1 | 6692923 | G | C | Missense | Benign | Tolerated |
| Paternal | ARHGAP29 | chr1 | 94670710 | T | C | Missense | Benign | Tolerated |
| Paternal | FLG2 | chr1 | 152328703 | T | C | Missense | Benign | Tolerated |
| Paternal | DDR2 | chr1 | 162735845 | C | A | Missense | Benign | Tolerated |
| Paternal | HLX | chr1 | 221053404 | G | A | Missense | Benign | Damaging |
| Paternal | RYR2 | chr1 | 237774273 | T | A | Missense | Damaging | Damaging |
| Paternal | ASTL | chr2 | 96803328 | G | A | Missense | Damaging | Tolerated |
| Paternal | RIF1 | chr2 | 152321732 | G | C | Missense | Benign | Damaging |
| Paternal | DPP4 | chr2 | 162873630 | C | T | Missense | Benign | Tolerated |
| Paternal | GBE1 | chr3 | 81720086 | G | A | Missense | PD | Damaging |
| Paternal | MYH15 | chr3 | 108179149 | T | C | Missense | Damaging | Damaging |
| Paternal | COL6A5 | chr3 | 130104070 | C | T | Missense | Damaging | Tolerated |
| Paternal | MUC4 | chr3 | 195507206 | T | C | Missense | Benign | NA |
| Paternal | MUC4 | chr3 | 195507982 | T | G | Missense | Benign | NA |
| Paternal | MUC4 | chr3 | 195515320 | G | T | Missense | Benign | NA |
| Paternal | BOD1L1 | chr4 | 13605705 | T | A | Missense | Damaging | Tolerated |
| Paternal | TMPRSS11A | chr4 | 68777178 | G | A | Missense | Damaging | Tolerated |
| Paternal | BMPR1B | chr4 | 96052611 | A | G | Missense | Benign | Tolerated |
| Paternal | CENPE | chr4 | 104072450 | C | T | Missense | Benign | Tolerated |
| Paternal | ITGA2 | chr5 | 52367836 | C | A | Missense | PD | Tolerated |
| Paternal | DMGDH | chr5 | 78365377 | G | T | Missense | Benign | Tolerated |
| Paternal | PDLIM7 | chr5 | 176918032 | G | A | Missense | PD | Damaging |
| Paternal | DSP | chr6 | 7585885 | T | C | Missense | Benign | Tolerated |
| Paternal | COL12A1 | chr6 | 75848275 | T | A | Missense | Damaging | Damaging |
| Paternal | AIM1 | chr6 | 107006389 | T | C | Missense | Damaging | Damaging |
| Paternal | KIAA0408 | chr6 | 127768100 | T | G | Missense | Benign | Tolerated |
| Paternal | TXLNB | chr6 | 139564289 | C | T | Missense | Damaging | NA |
| Paternal | PLEKHG1 | chr6 | 151161581 | A | C | Missense | Benign | Tolerated |
| Paternal | GLI3 | chr7 | 42005564 | G | A | Missense | Benign | Tolerated |
| Paternal | CCL24 | chr7 | 75441248 | C | T | Missense | Benign | Tolerated |
| Paternal | MUC17 | chr7 | 100676729 | A | G | Missense | Benign | Tolerated |
| Paternal | SRPK2 | chr7 | 104783533 | T | C | Missense | Benign | Tolerated |
| Paternal | OR9A4 | chr7 | 141618815 | TTGTC | T | Fs del | NA | NA |
| Paternal | MTUS1 | chr8 | 17504542 | T | C | Missense | Damaging | Damaging |
| Paternal | ZNF16 | chr8 | 146171528 | G | C | Missense | Benign | Tolerated |
| Paternal | SPATA31A3 | chr9 | 40706227 | G | A | Missense | PD | Tolerated |
| Paternal | SPATA31D1 | chr9 | 84607342 | C | T | Missense | Benign | Tolerated |
| Paternal | SLC28A3 | chr9 | 86894294 | A | G | Missense | Damaging | Damaging |
| Paternal | TMEM203 | chr9 | 140099542 | T | C | Missense | Benign | Tolerated |
| Paternal | CREM | chr10 | 35467878 | A | G | Missense | Damaging | Damaging |
| Paternal | FAM21A | chr10 | 51889757 | TAGTC | T | Fs del | NA | NA |
| Paternal | CRTAC1 | chr10 | 99683135 | C | A | Missense | Damaging | Damaging |
| Paternal | CCDC186 | chr10 | 115922443 | T | A | Missense | Benign | Tolerated |
| Paternal | OR56B4 | chr11 | 6129502 | T | G | Missense | Damaging | Damaging |
| Paternal | ROM1 | chr11 | 62381078 | G | A | Missense | Benign | Tolerated |
| Paternal | METTL12 | chr11 | 62434033 | G | A | Missense | Benign | Tolerated |
| Paternal | TRIM49C | chr11 | 89769108 | A | G | Missense | Benign | NA |
| Paternal | GALNT8 | chr12 | 4835837 | ACT | A | Fs del | NA | NA |
| Paternal | KCNC2 | chr12 | 75601499 | C | T | Missense | Benign | Tolerated |
| Paternal | SACS | chr13 | 23911921 | A | G | Missense | Benign | Tolerated |
| Paternal | JMJD7-PLA2G4B, PLA2G4B | chr15 | 42138418 | C | T | Missense | Benign | Tolerated |
| Paternal | SPATA5L1 | chr15 | 45694658 | G | A | Missense | Benign | Tolerated |
| Paternal | FAM227B | chr15 | 49800520 | C | A | Missense | Damaging | Damaging |
| Paternal | CRAMP1L | chr16 | 1719041 | G | A | Missense | Benign | Tolerated |
| Paternal | VASN | chr16 | 4431819 | G | A | Missense | Benign | Tolerated |
| Paternal | ST3GAL2 | chr16 | 70432128 | C | A | Missense | Benign | Tolerated |
| Paternal | BCAR1 | chr16 | 75267749 | G | C | Missense | Damaging | Tolerated |
| Paternal | GSE1 | chr16 | 85690160 | G | A | Missense | Benign | Tolerated |
| Paternal | KIF1C | chr17 | 4927349 | G | A | Missense | Benign | Tolerated |
| Paternal | ZNF286A | chr17 | 15619863 | A | T | Missense | Benign | Tolerated |
| Paternal | CCDC144A | chr17 | 16623864 | G | A | Missense | PD | Tolerated |
| Paternal | LIG3 | chr17 | 33310367 | G | A | Missense | Damaging | Damaging |
| Paternal | KLHL10 | chr17 | 40004260 | A | G | Missense | Benign | NA |
| Paternal | SKOR2 | chr18 | 44773552 | T | G | Missense | NA | Tolerated |
| Paternal | MBD1 | chr18 | 47801759 | A | T | Missense | PD | Tolerated |
| Paternal | SIRT6 | chr19 | 4174772 | G | A | Missense | Benign | Tolerated |
| Paternal | BEST2 | chr19 | 12863428 | C | T | Stopgain | NA | Tolerated |
| Paternal | PTGIR | chr19 | 47124820 | A | T | Missense | Damaging | Damaging |
| Paternal | SYNGR4 | chr19 | 48879571 | A | G | Missense | Benign | Tolerated |
| Paternal | AKT1S1 | chr19 | 50373209 | T | C | Missense | Damaging | Tolerated |
| Paternal | CDC42EP5 | chr19 | 54976323 | TG | T | Fs del | NA | NA |
| Paternal | ACSS1 | chr20 | 25028764 | A | C | Missense | Damaging | Damaging |
| Paternal | FAM209A | chr20 | 55099895 | C | G | Missense | Benign | Tolerated |
| Paternal | SLC17A9 | chr20 | 61588250 | G | A | Missense | PD | Damaging |
| Paternal | MGAT3 | chr22 | 39883831 | G | A | Missense | Benign | Tolerated |
| Paternal | ADSL | chr22 | 40742633 | C | T | Missense | Benign | Damaging |
| Paternal | ARSA | chr22 | 51066183 | G | A | Missense | Benign | Tolerated |

Chr, chromosome; Fs del, frameshift deletion; Ref, reference allele; Var, variant allele; PD, probably damaging; NA, not available.
